# The Chloroplast Epitranscriptome: Factors, Sites, Regulation, and Detection Methods

**DOI:** 10.3390/genes12081121

**Published:** 2021-07-24

**Authors:** Nikolay Manavski, Alexandre Vicente, Wei Chi, Jörg Meurer

**Affiliations:** 1 Plant Molecular Biology, Faculty of Biology, Ludwig-Maximilians-University Munich, Großhaderner Street 2-4, 82152 Planegg-Martinsried, Germany; n.manavski@biologie.uni-muenchen.de (N.M.); a.vicente@campus.lmu.de (A.V.); 2 Photosynthesis Research Center, Key Laboratory of Photobiology, Institute of Botany, Chinese Academy of Sciences, Beijing 100093, China; chiweimr@ibcas.ac.cn

**Keywords:** chloroplast, RNA metabolism, epitranscriptome, RNA methylation, posttranscriptional regulation, development, acclimation, stress response

## Abstract

Modifications in nucleic acids are present in all three domains of life. More than 170 distinct chemical modifications have been reported in cellular RNAs to date. Collectively termed as epitranscriptome, these RNA modifications are often dynamic and involve distinct regulatory proteins that install, remove, and interpret these marks in a site-specific manner. Covalent nucleotide modifications-such as methylations at diverse positions in the bases, polyuridylation, and pseudouridylation and many others impact various events in the lifecycle of an RNA such as folding, localization, processing, stability, ribosome assembly, and translational processes and are thus crucial regulators of the RNA metabolism. In plants, the nuclear/cytoplasmic epitranscriptome plays important roles in a wide range of biological processes, such as organ development, viral infection, and physiological means. Notably, recent transcriptome-wide analyses have also revealed novel dynamic modifications not only in plant nuclear/cytoplasmic RNAs related to photosynthesis but especially in chloroplast mRNAs, suggesting important and hitherto undefined regulatory steps in plastid functions and gene expression. Here we report on the latest findings of known plastid RNA modifications and highlight their relevance for the post-transcriptional regulation of chloroplast gene expression and their role in controlling plant development, stress reactions, and acclimation processes.

## 1. Introduction

The chloroplast is the result of an endosymbiotic event in which a cyanobacterium was ingested by a eukaryotic host cell. Although chloroplast biogenesis requires regulation of transcription rates, umpteen posttranscriptional events predominate in the development- and environment-dependent control of gene expression [1,2,3,4,5]. Chloroplasts have retained parts of the cyanobacterial-derived translation and RNA degradation system, such as masking of RNAs by polyadenylation, but only few exoribonucleases with little sequence specificity act on chloroplast RNA species [6]. Unlike in cyanobacteria, in plant chloroplasts nearly all polycistronic precursor transcripts are processed by endonucleases and resulting products are further stabilized and subjected to additional enzyme-based modifications [3,7,8,9,10]. This necessitated the recruitment of often plant-specific and nucleus-encoded proteins, which enable regulation of chloroplast gene expression on single gene levels [2,4,11]. Little is known about factors that authentically regulate plastid mRNA stability and/or translation and even less about the posttranscriptional control meditated by metabolic processes or endogenous and external stimuli [2,11,12,13]. The role of the plant epitranscriptome in overcoming the challenges of plant life has only just begun to be studied in depth in the different genetic compartments.

Recently, methodological advances in next-generation sequencing including nanopore sequencing, CLIP-technologies, antibody-based approaches, mass spectrometry, ribosomal profiling, as well as the use of chemical and enzymatic modifications have significantly increased our knowledge about epitranscriptomics and laid the foundation for understanding its role in regulation of gene expression in prokaryotes and eukaryotes [14].

More than 170 different modifications in coding and non-coding RNAs as well as several hundred factors involved in epitranscriptomics have been discovered [15,16,17]. The majority of the modifications are found in the bases of predominantly non-coding and coding RNAs and only a few in the phosphate or sugar backbone. While our knowledge about RNA modifications in the plant nuclear/cytoplasmic system is considerable rich, little attention has been paid to the chloroplast epitranscriptome so far [16,18,19,20,21]. As in other systems, chloroplast transcripts potentially provide multiple platforms for numerous RNA modifiers (writers and erasers) and interpreters (readers). The latter recognize the modifications presumably reflecting a complex interplay between epitranscriptome players, RNA processing, and translation and thus important parts of the chloroplast metabolism and photosynthesis. Studying the response of these readers to endogenous and external stimuli in terms of RNA binding and the readout of modifications will significantly contribute to our understanding of the coordination of plastid gene expression and metabolism beyond the mere change in RNA levels. This is especially relevant since chloroplasts are believed to function as central sensors that perceive environmental signals in order to trigger plant gene expression and most likely the organellar epitranscriptome [22].

**Figure 1:**
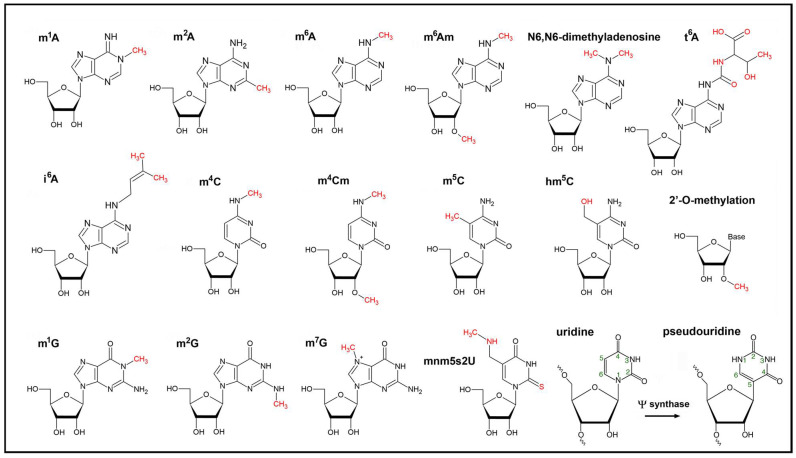
Chemical formulas of modified nucleotides found within chloroplast RNAs. The epitranscriptomic modifications are catalyzed by writers and are highlighted in red. The isomerization of uridine to pseudouridine (lower right) within RNAs is catalyzed by the Ψ synthase. The extra hydrogen bond at position N1 of pseudouridine stabilizes base pairing and RNA structure.

While the role of polyadenylation in RNA degradation and mostly C to U editing events in chloroplasts are well understood [23], little is known about the nature, dynamics, and functions of other modifications such as the diverse methylation steps m^6^A, m^1^A, m^7^G, m^5^C or hm^5^C, adenosine dimethyltransfer (m^6^Am) [9] as well as uridylation, pseudouridylation, removal of the noncanonical NAD^+^ cap, the biosynthesis of 5-methylaminomethyl-2-thiouridine (mnm(5)s(2)U) of tRNAs, and many others (Figure 1). All their functions are embedded in the transition from RNAs to proteins and are most likely important for the regulation of RNA localization, structure, stability, processing, ribosome assembly, and translational events. Modifiers and readers are believed to play important roles especially for abiotic stress responses, plant acclimation, and developmental processes [24].

Our knowledge about the function of chloroplast epitranscriptomic activities is still in its infancy. Forthcoming state-of-the-art ‘omics’ technologies will considerably improve the monitoring of RNA modification networks at the genomic scale in the plant genetic compartments and will certainly shed light on the function and regulation of epitranscriptomic players. In this review, we discuss our present knowledge about novel methods for the identification of RNA modifications, bioinformatic tools, and the potential physiological roles of RNA modifiers and interpreters in plant nuclear/cytoplasmic gene expression related to photosynthesis and the post-transcriptional fate of chloroplast RNAs.

## 2. Methods for the Detection of RNA Modification Marks

Immunoprecipitation of modified RNAs in combination with high-throughput sequencing, mass spectrometry-based techniques, and nanopore sequencing are the three most common approaches for the transcriptome-wide detection and quantification of epitranscriptomic marks. Some of the methods are further combined with chemical or enzymatic steps to identify signature-based marks in RNA molecules [25]. However, it is important to consider as mentioned below that each system has its intrinsic strengths and limitations.

### 2.1. Antibody-Based Approaches and Next-Generation Sequencing

Since antibodies are available for m^1^A, m^6^A, and m^5^C and other base modifications, several studies of the cellular epitranscriptome in Arabidopsis focused on immunological approaches, such as the predominant N^6^A-methylated RNA immunoprecipitation sequencing (MeRIP/m^6^A-seq). This technique requires significant quantities of RNAs, high quality antibodies with little cross reactivity, and involves immunoprecipitation of about ~100 nucleotides-long RNA fragments using m^6^A-specific antibodies, followed by sequencing of the immunoprecipitated fragments or the corresponding cDNAs [26]. However, this approach has several limitations with respect to the resolution and the specificity of the antibody. For example, antibodies for m^6^A can potentially also detect a second base modification, N^6^,2-O-dimethyladenosine (m^6^Am), which is located at the 5’ end of transcripts. In addition, the mandatory fragmentation of RNAs for library constructions may result in the underrepresentation of m^6^A sites [27]. To overcome these drawbacks, new approaches have been developed [28,29,30,31,32]. For example, m^6^A individual-nucleotide-resolution cross-linking and immunoprecipitation (miCLIP) can be used to induce specific mutational signatures that allow for the precise identification of m^6^A residues in RNA molecules. In this approach, antibodies raised against m^6^A are UV-crosslinked to RNAs and subsequent reverse transcription of crosslinked RNAs results in a precise pattern of mutations or truncations in the cDNA. These signatures are computationally identified and allow mapping of m^6^A residues at single-nucleotide resolution [33].

Sequencing approaches in epitranscriptomics rely on cDNAs as template. A weak point of this method is that some of the epitranscriptomic information is lost when immunoprecipitated RNA is reverse transcribed. However, some of the modification marks, such as m^1^A, m^3^C, and m^1^G, can easily be detected because they induce reverse transcription errors or termination, as compared to their unmodified sites. For the detection of m^5^C, RNA can also be treated with sodium bisulfite prior reverse transcription. This chemical compound deaminates cytosine to uracil resulting in a thymine during reverse transcription. In contrast to the unmethylated cytosine, the m^5^C methylation is not prone to this deamination and thus can be detected.

### 2.2. Mass Spectrometric Approaches

The prerequisite for classical mass spectrometry in the detection of diverse RNA modifications including m^6^A is based on the complete enzymatic digestion of the RNA into individual nucleotides or nucleosides followed by various LC–MS/MS (liquid chromatography coupled to tandem mass spectrometry)-based methods [25]. After ionization the ions are detected based on their mass-to-charge ratio (*m*/*z*) to estimate their molecular mass and abundance. The modified RNA digestion products can readily be identified due to an increased molecular mass compared with the unmodified standards. This method plays an important role in the discovery, simultaneous detection, and quantification of many different RNA modifications. However, despite its high sensitivity, accuracy, and low detection limit in the fmol to amol range, co-eluting compounds with the identical masses, such as the modifications m^1^G and m^2^G, cannot be distinguished. Importantly, due to the complete digestion all information about the sequence context and co-occurrence of modifications is entirely lost. To overcome this shortcoming, RNA molecules are partially digested using specific RNases. The resulting RNA oligonucleotides are then analyzed by MS/MS and the covalent modifications are recognized with single-nucleotide resolution based on the molecular mass shift. A comparison with mass spectra provided by a sequence database enables the determination of the precise position and nature of modifications within the oligonucleotide that can be determined using a computational platform for high-throughput data mining [34].

### 2.3. Nanopore Sequencing

Nanopore direct RNA sequencing (DRS) can be applied to measure and quantify many but not all RNA methylations at defined positions without fragmentation or amplification. This technology relies on a protein nanopore that resides in a membrane through which an electrical current is created. The RNA sequence can be identified by the magnitude of signals transmitted when intact RNAs pass through the nanopore by a motor protein [35]. The use of nanopore sequencing was also successfully applied in revealing full-length mRNAs, mapping of the 5’ cap, the position, and estimated length of the poly(A) tail, different patterns of alternative splicing, and sites of internal RNA cleavage in Arabidopsis. Moreover, novel examples of intronic alternative polyadenylation that potentially modulates gene functions were identified using this technique.

## 3. The Cellular m^6^A RNA Epitranscriptome

### 3.1. m^6^A Methylation of Nuclear-Derived RNAs Related to Chloroplast Functions

**Figure 2:**
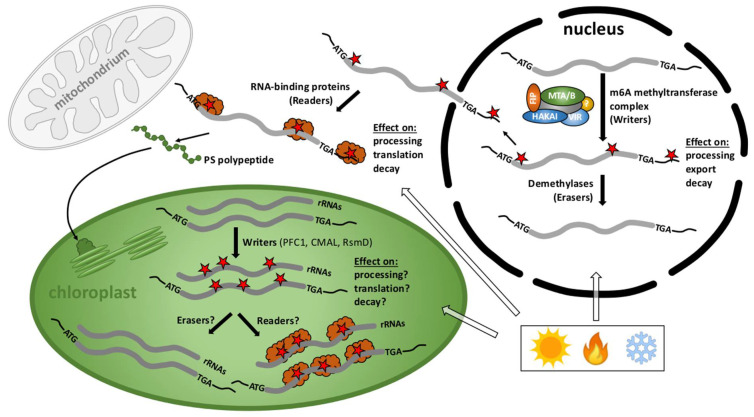
Cellular scenario of the RNA methylome in plants. m^6^A methylation and demethylation of nuclear-derived RNAs mainly takes place in the nucleus close to start and stop codons and in the 3’ UTRs and participate in the processing, stability, and localization of RNAs whereas m^6^A readers function mostly in the cytoplasm. m^6^A methylated transcripts predominantly encode chloroplast proteins important for gene expression, photosynthesis, and other plastid functions. Only three 16S rRNA writers–PFC1 for m^6^A, CMAL for m^4^Cm, and RsmD for m^2^G methylations-have been described in chloroplasts but writers for mRNAs, as well as erasers and readers are entirely unknown. The activity of these modifiers and interpreters is presumably crucial for the fate of transcripts important for plant development, stress responses, and acclimation processes upon environmental changes. Red stars: RNA methylation marks; orange clouds: readers.

Recent data revealed that m^6^A methylation marks in nuclear-derived RNAs are often related to chloroplast function, thus we will provide a brief overview of the nuclear/cytoplasmic m^6^A methylome with a focus on this organelle (Figure 2). With respect to RNA methylation, such as the most widespread adenosine methylation at the N6 position (m^6^A), the mainly nuclear localized modifiers (methyltransferases and demethylases) and mostly cytoplasmic interpreters are called writers, erasers, and readers, respectively [36]. The nuclear m^6^A writer complex consists of several conserved and essential components in eukaryotes, including the methyltransferases MTA and MTB, the splicing factor FKBP12 Interacting Protein37 (FIP37), VIRILIZER (VIR), and the conserved E3 ubiquitin ligase HAKAI [37]. DRS and miCLIP were recently applied to examine the epitranscriptome of the leaky *vir-1* mutant defective in nuclear m^6^A methylation and VIR-complemented lines in Arabidopsis [32]. Frequent cleavage and polyadenylation of the mRNA encoding a chloroplast envelope-bound plant homeodomain transcription factor (PTM) with transmembrane domains was found. PTM (AT5G35210) resides in the outer chloroplast membrane and was suggested to be involved in retrograde signaling upon cleavage of a C-terminal transmembrane domain that sequesters it to the chloroplast [32,38]. Cleavage and polyadenylation of the *ptm* intron 10 terminates transcription prior to a sequence encoding the transmembrane domain, consequently bypassing established retrograde control [32]. 17,491 sites with restored m^6^A modifications in the VIR-complemented line were identified. The AAm^6^ACU and AAm^6^ACA motifs were confirmed to be the most frequently detected m^6^A marks in Arabidopsis [39,40,41]. Several sites associated with the motif AGm^6^AUU were also detected, raising the possibility that a C following m^6^A is not a constant feature of the Arabidopsis m^6^A code [32]. The preferential 3’ UTR localization of m^6^A in cytoplasmic mRNAs was also confirmed. The differential error sites were exclusively found in this region and no enrichment over stop codons was identified using both, miCLIP or DRS. Strikingly, the impact of m^6^A loss on pre-mRNA processing was determined and a clear defect in RNA 3’ end formation in *vir-1* was observed [32]. 3579 genes with an altered 3’ position profile in the *vir-1* mutant were identified. For instance, the *prpl34* mRNA, which encodes a chloroplast ribosomal protein, is methylated in at least two positions in the 3’ UTR and displays an increase in alternative polyadenylation at a proximal poly(A) site in the *vir-1* mutant. These findings suggest that changes in 3’ end poly(A) position of RNAs in the *vir-1* mutant may result directly from the loss of m^6^A and implies a crucial role of the cellular m^6^A methylome in plastid gene expression.

Conflicting results in the Arabidopsis epitranscriptome, such as the enrichment of the m^6^A distribution within the plant mRNA molecules, can be solved using state-of-the-art approaches and new technologies. Still, it is important to restate that the effects of m^6^A are challenging and possibly condition-dependent. To date, many chloroplast-related, nucleus encoded transcripts that carry m^6^A were identified (Supplementary Table S1). Regardless of nucleus- or chloroplast-derived transcripts, a compelling connection between m^6^A RNA modifications and chloroplast and/or acclimation functions in plants seems to be clear. Despite the obvious progress summarized here, the exact understanding of how m^6^A modifications regulate the function of nucleus-encoded RNAs that encode chloroplast proteins remains a future challenge (Figure 2).

In general, the m^6^A modification is subjected to dynamic regulation in both development and response to cellular stimuli and ever-changing conditions in eukaryotes [25,42]. Although m^6^A appears to be the most abundant internal RNA modification of plants, the m^6^A pattern and its regulation in humans is by far much better investigated than in plants [37]. Importantly, a relatively high proportion of nuclear/cytoplasmic transcripts encoding photosynthesis-related proteins have been shown to undergo m^6^A modifications implying important roles in chloroplast functions. The m^6^A modifications generally play critical roles in many areas of the plant life [42,43]. Similar to the animal system the m^6^A:A ratio is 1.5% in young Arabidopsis seedlings [44]. Elucidation of the function of the m^6^A RNA modification is a challenging and growing field in plant RNA research [45]. Rapidly evolving methodological approaches will allow us to increase our understanding about the function and regulation of m^6^A in plants, which certainly will contribute to improve our knowledge about cellular functions, developmental cycles, and acclimation processes related to chloroplast functions.

The m^6^A methylome in plants was first identified in two accessions of *A. thaliana*, Can-0 and Hen-16, two wild-collected lines from areas that vary drastically in photosynthetically active radiation values. However, the m^6^A modifications were shown to be remarkably conserved across these two lines. Surprisingly, m^6^A was enriched not only within the 3’ UTRs and stop codons but also around the start codons, a feature only observed in plant RNAs [39]. The consensus recognition sequence of nuclear/cytoplasmic transcripts has been described as RRm^6^ACH in the epitranscriptome of mammals, where R = G > A and H = U > A > C [26,46]. Interestingly, in plant RNAs new conserved motifs were found, indicating the presence of distinct target sequence motifs for m^6^A target-methylations [21,39]. For example, the URUAY (R = G > A,Y = U > A) m^6^A methylation motif is plant specific and was shown to have a role in RNA stabilization [47]. In both, Can-0 and Hen-16, gene ontology unveiled many biological pathways related to chloroplast functions. In particular, more than 60% of cytoplasmic transcripts containing m^6^A in both, start and stop codons and about 40% of those carrying the modification only in the start site were highly associated with photosynthetic functions. A complete list can be accessed in a previous work [39].

Differential m^6^A patterns of cytoplasmic transcripts across different organs were also investigated in Arabidopsis [40]. More than 70% of the transcripts were m^6^A modified in leaves, flowers, and roots. The consensus sequence RRm^6^ACH was found in over 75% of the transcripts, but only one dominant peak of m^6^A enrichment was identified around the 3’ UTR and stop codons in the Arabidopsis transcriptome. Notably, all three organs analyzed share about 290 m^6^A-methylated transcripts and their coding proteins are mostly located in the chloroplast. Most interestingly, differential m^6^A methylation among leaves, flowers, and roots showed that green leaves had the highest extent of m^6^A methylation among the three organs. These transcripts are mainly related to photosynthesis, regulation of transcription, and stress response (Supplementary Table S1). Highly methylated transcripts presented in leaves and roots had specific functions related to the respective organs—photosynthesis in leaves and transport in roots [40].

The extent of m^6^A methylation was also compared to the levels of the respective transcripts in three organs of Arabidopsis. Most of the highly expressed transcripts were less modified by m^6^A when compared with transcripts expressed at low level. This observation implies an important function of m^6^A in regulation of RNA levels and/or stability in plant cells. Low level transcripts may require a relatively higher extent of m^6^A modifications to maintain RNA stability in the cells and vice versa [40]. A role of m^6^A in the stabilization of mRNAs under salt stress has been reported in Arabidopsis. In this case, m^6^A is dynamically added to salt-stress-related transcripts to protect RNAs from degradation [48]. The process of flowering, for instance, is delayed in *alkbh10b* mutant plants, which lack an m^6^A eraser. This phenotype can be explained by the destabilization of transcripts involved in flowering transition when m^6^A modifications are not reversed [49]. Therefore, the exact mechanisms of regulation involving m^6^A in plants are far from being identified. Why under certain conditions m^6^A stabilizes or destabilizes a specific transcript is still to be determined. So far, the dependence of the stability of nuclear-encoded RNAs associated with plastid functions also remains an open question. Whether and how m^6^A methylation in the nucleus/cytoplasmic system reshapes the proteome or modulates gene expression within the chloroplast and vice versa remains elusive.

### 3.2. General Aspects on m^6^A Methylation Marks in Chloroplast RNAs

Virtually nothing is known about m^6^A epitranscriptome players in chloroplasts. In contrast to nuclear/cytoplasmic systems, only one chloroplast m^6^A RNA writer has been described [50] but erasers and readers are yet to be discovered (Figure 2). The m^6^A methylome was studied in Arabidopsis chloroplasts and mitochondria [42,51]. mtRNAs in both Arabidopsis and cauliflower undergo N6-adenosine methylation modifications with an occurrence of about 4–5 m^6^A sites per 1000 adenosine residues. Several m^6^A modifications were detrimental for translation, while a single modification in the start codon suggested an enhancement in the translatability of the mitochondrial transcript [51].

Remarkably, chloroplast transcripts are highly m^6^A methylated, implying important roles in photosynthesis and/or plastid gene expression [42,43]. Over 98% of chloroplast transcripts were chemically modified by m^6^A, which is by far much more than the modification status found in the nuclear transcriptome (73%). Furthermore, about 4.6 to 5.8 m^6^A sites per transcript were found in the chloroplast but only about 1.4 to 2.0 sites per transcript in the cytoplasm, again emphasizing an important link between m^6^A modifications and chloroplast functions [41]. The most modified transcripts found in this analysis were associated with chloroplast rRNA and mRNAs (Supplementary Table S1).

The previously observed dominant m^6^A enrichment within the 3´ UTR and near stop codons in nuclear-derived mRNAs was not observed in the chloroplast. Instead, m^6^A peaks were found evenly distributed in chloroplast transcripts with higher methylations in exons when compared to introns, suggesting that the regulatory mechanisms may be different between the nucleus/cytoplasm and chloroplast systems. The translation and stability of chloroplast transcripts are commonly regulated by factors acting on 5’ and 3’ UTRs. In addition, degradation of plastid RNAs is thought to be initiated by endonucleolytic cleavages [4,52]. Thus, it is likely that m^6^A methylation in conjunction with RNA-binding proteins controls the fate of mRNAs in the plant organelle at multiple levels.

Most surprisingly, many m^6^A consensus sequences in the chloroplasts and mitochondria share homology to those in mammals and plant nuclear transcritptomes, indicating an evolutionary related process [41,51]. The two most common sequence motifs found in the Arabidopsis chloroplast transcriptome were GGm^6^ACC and GGm^6^ACU. The methylation extent compared to RNA levels in the chloroplast was similar to that found in the nuclear-derived RNAs in Arabidopsis, as most of the highly expressed plastid transcripts were less modified by m^6^A, and vice versa, corroborating a related or analogous development. However, tissue-specific deviations were also observed. For example, in root amyloplasts the moderately expressed transcripts were more methylated and those expressed at lower or higher levels carried less m^6^A modifications [41].

## 4. tRNA Modifications

tRNAs are vital components of the translation machinery and more than 100 tRNA modifications are known so far, which all seem to be associated with translation efficiency, accuracy, and preventing ribosomal frameshifting. Modifications are often conserved and occur at specific sites of distinct chloroplast tRNAs, such as m^7^G at tRNA(Met) [53], i^6^A and m^1^G at position 37 of tRNA(Cys) [54], t^6^A, m^2^G and m^7^G at tRNA(Ile) [55], m^7^G at tRNA(Leu) [56] and m^2^A at tRNA(Met) [57], pseudouridylation (see below), and many others. Furthermore, hypermodifications in anti-codon loops are often important for decoding the genetic code. A number of studies have shown that tRNA modifications influence various developmental processes and functions in the stress response in distinct organisms, but little attention has been drawn to the role of diverse tRNA modifications in chloroplasts [58].

### 4.1. 5-Methylaminomethyl-2-Thiouridine Modification

To read all possible 64 triple codons, a minimum of 32 tRNAs is needed. However, plant plastids contain less than 32 distinct tRNAs, suggesting that tRNAs with U in their wobble position might pair with any of the four bases at the third position of the codon via superwobble [59]. The base pairing selectivity at the wobble position, that is to say the stringency or flexibility of the anticodon of tRNAs, is regulated by posttranscriptional modifications of the wobble U (U34) and has been shown to be crucial for correct and efficient translation [60,61,62] as well as for preventing ribosomal frameshifting [63]. One of these wobble nucleoside modifications is the 5-methylaminomethyl-2-thiouridine or mnm5s2U.

The bacterial glucose-inhibited division (*gid*) operon encodes the two GidA and GidB enzymes essential for the biosynthesis of mnm5s2U of tRNAs and the S-adenosyl-L-methionine (SAM)-dependent methylation of the 16S rRNA in the highly conserved 530 loop important for ribosomal function, respectively [64]. Mutant analysis has shown that GidA and GidB activities are important for stress response, growth, morphology, antibiotic resistance, and bacterial pathogenesis [64].

In *E. coli* for example, the mnm5s2U modification is a two-step process, in which U34 of the tRNA is first modified to 5-carboxymethylaminomethyl-2-thiouridine (cmnm5s2U) by TrmE (also known as tRNA modification E MnmE) together with GidA [65,66] and then decarboxylated to nm5s2U and methylated in a S-adenosyl-L-methionine- (SAM)-dependent manner to produce mnm5s2U by the enzyme TrmC (also called MnmC) [67]. GidA and the GTP-binding protein MnmE, form a heterotetrameric α2β2 complex consisting of two homodimers in order to bind and to interdependently modify particular tRNAs at the wobble uridine base U34 of the first anticodon position in FAD-and GTP-dependent reactions to form mnm5s2U [68].

Near-isogenic lines generated by introgression and mutants in rice exhibited a pleiotropic phenotype with reduced chloroplast protein levels and altered gene expression that highly affects retrograde signaling [69]. Map-based cloning revealed that the allele PLEIOTROPIC DEVELOPMENTAL DEFECTS (PDD) is responsible for the phenotype and closely related to plant and cyanobacterial TrmE proteins. PDD is preferentially expressed in photosynthetic tissue. As in bacteria, the rice homolog is able to form dimers in vivo and to exhibit GTPase activity [69]. The natural variation form of PDD, called *PDD^OL^*, containing a 6-bp deletion as well as 28 SNPs in its ORF, was incapable of forming homodimers in introgression lines and showed a reduced GTPase activity. NIL-*PDD^OL^* plants showed multiple developmental defects accompanied by decreased levels of proteins involved in photosynthesis and ribosome biogenesis. Investigations of the tRNA modifications by LC-MS/MS revealed that modification levels of mnm5s2U were significantly reduced in NIL-*PDD^OL^* as compared to the WT.

Moreover, altered chloroplast transcription and translation in PDDOL seems to activate retrograde signaling, as expression levels of the photosynthesis-associated nuclear genes were significantly reduced in *PDD^OL^*. While the function and biosynthesis of the mnm5s2U modification in bacteria seems to be relatively well understood, the roles of mnm5s2U in chloroplasts remains elusive.

It is very interesting that natural variations of a chloroplast tRNA-modifying enzyme led to severe pleiotropic defects that not only function in chloroplast biogenesis but also in plant development and demonstrates a fast-evolving plastid RNA metabolism despite its conserved mechanism [4]. Whether the plant TrmE also forms a heterotetramer in association with a GidA homolog remains to be shown. Remarkably, the Arabidopsis genome encodes a highly conserved GidA homolog (AT2G13440) which is considered to be located in chloroplasts when using several prediction servers (aramemnon.uni-koeln.de, accessed on 23.07.2021). However, a functional characterization and experimental investigations of the capability of the GidA homolog to form functional tetrameric complexes with the plant TrmE are lacking. Addressing these points in future studies will certainly contribute to improve our understanding of mnm5s2U in chloroplasts.

### 4.2. i^6^A Modification

The 37th base of a subset of tRNAs next to the anticodon can be modified by bulky additions including N6-isopentenyl adenosine (i^6^A) in all kingdoms of life. The i^6^A modification can be modified further to 2-methyl-thio-N6-isopentenyladenosine (ms2i^6^A) and is believed to stabilize the Watson-Crick base pairing by base stacking. The isopentenylation is catalyzed by a conserved isopentenyl transferase important for translation accuracy, efficiency, and non-sense suppression [70].

The isopentenylation is also conserved at position 37 of chloroplast cysteine tRNAs carrying a GCA anticodon. It has been shown that the chloroplast tRNA^Cys^ isoacceptor at position 37 stimulates read-through over UGA stop codons and thus has been considered as a natural UGA stop codon suppressor [54].

## 5. Plastid rRNA Methylations

### 5.1. m^5^C Modifications of Nuclear-Derived mRNAs, tRNAs, and Plastid rRNAs

5-methylcytosine (m^5^C) is a well-characterized RNA modification which has been found in high amounts in abundant RNAs in both prokaryotes and eukaryotes (Figure 1). The m^5^C marks in plant RNAs are at least a factor of 10 lower than m^6^A marks and enriched in coding regions of mRNAs where they play an important role in translation efficiency in Arabidopsis [71]. The recently discovered nuclear methyltransferase OsNSUN2 in rice is responsible for approximately 30% of m^5^C modifications of nuclear transcripts which depends on heat. Thus, OsNSUN2 is the most important m^5^C methyltransferase and stimulates the translation of those cytoplasmic mRNAs that are important for the maintenance of chloroplast functions and the acclimation response to heat [72].

The m^5^C sites in tRNAs commonly occur in the variable region and anticodon loop and are generally required for tRNA stability and efficient translation [73,74,75]. By combining RNA bisulfite conversion with second generation Illumina sequencing (RBS-seq), Burgess et al., (2015) [76] identified a total of 39 highly methylated m^5^C sites in predicted structural positions of tRNAs from Arabidopsis. Although m^5^C has been found to occur on distinct tRNAs and rRNAs in many organisms [76], no m^5^C sites were identified in plastid and mitochondrial mRNAs or tRNAs so far [76,77]. Considering the eubacterial origin of mitochondria and chloroplasts, this finding is not surprising given that m^5^C sites were not associated with tRNAs in bacteria [24].

m^5^C sites play important roles in rRNA processing, structure, and translation [78,79,80]. Unlike tRNAs, three m^5^C sites were identified in plastid rRNAs: C916 in 16S as well as C1940 and C1977 in 23S rRNAs. While C916 in 16S and C1977 in 23S rRNA are conserved between bacteria and plastids, C1940 in the 23S rRNA seems to be unique to plastids, suggesting that it has derived newly in the green endosymbiont. While m^5^C sites have been identified in plastid rRNAs, the RNA methyltransferases in charge of the formation of m^5^C remain to be identified. In *E. coli*, there are three RNA methyltransferases accounting for the methylcytosine modification: RsmB, RsmF, and RlmI. Recent plastid proteome analysis identified a putative tRNA-specific methyltransferase 4E (TRM4E; AT3G13180) within chloroplasts [81,82]. TRM4E displays a considerable similarity to RsmB, raising the possibility that TRM4E might be the methyltransferase responsible for the methylcytosine modification of plastid rRNAs.

### 5.2. m^4^Cm Modification of the Plastid 16S rRNA

At the P-site of the 16S rRNA of *E. coli* there is a unique dimethyl modification, *N*^4^, 2′-*O*-dimethylcytidine (m^4^Cm) located at position 1402, which was discovered more than half a century ago. m^4^Cm1402 plays a role in fine-tuning the shape and function of the P-site, thus increasing decoding fidelity. Two distinct SAM-dependent RNA methyltransferases *mraW* (*rsmH*) and *yraL* (*rsmI*) are responsible for the *N*^4^ and 2′-*O*-methylations of C1402, respectively. The dimethyl modification of C1402 is conserved in bacteria and plastids [5]. Using bisulfate sequencing, the N4-methylation of C1352 in the plastid 16S rRNA of Arabidopsis was detected, which corresponds to C1402 of *E. coli*. This methylation is accomplished by the conserved chloroplast ortholog of RsmH, called CMAL [5]. CMAL is important for efficient chloroplast translation and presumably for controlling translational fidelity (Figure 2). Although CMAL orthologs and the methylation sites are conserved, in contrast to bacteria the knockout of CMAL in Arabidopsis impairs the chloroplast ribosome accumulation, which was not found in bacterial orthologs. The finding that loss of rRNA modifications in the decoding center can affect the biogenesis of the small subunit was also reported in yeast cells, suggesting that rRNA modifications can influence both ribosome synthesis and function in synergistic ways [83]. CMAL-mediated methylation may facilitate the structural rearrangements of helix 44 where C1352 is located in the 16S rRNA to establish a functionally optimal conformation during 30S ribosome assembly. This indicates additional functions of CMAL in plants. Interestingly, the loss of CMAL leads not only to defects in chloroplast function but also to abnormal leaf and root development and overall plant morphology. Further investigations showed that CMAL’s involvement in plant development is most likely due to its ability to modulate auxin-derived signaling pathways. Since auxin is a regulator for the cold-stress response, CMAL could also be involved in cold acclimation. It has been proposed that CMAL acquired novel functions in ribosome assembly and plant acclimation [5]. This idea is further supported by the observation that CMAL expression is downregulated in the cold. Thus, CMAL could represent a negative regulator for cold acclimation.

### 5.3. Dimethylation of the Plastid 16S rRNA

The KsgA gene in *E. coli* encodes an rRNA adenine dimethyltransferase responsible for the specific dimethylation at the N-6 position of the base moiety of two adjacent adenosines, A1518 and A1519 (m_2_^6^A_1518_m_2_^6^A_1519_), located near the 3′-end of the 16S rRNA [84]. This rRNA modification along with the enzyme involved in the methylation of the two nucleotides are well conserved in both eubacteria and eukaryotes. For example, DIM1, the yeast homolog of KsgA, was able to dimethylate the above-mentioned adenosines in the *E. coli* 16S rRNA and thus to complement KsgA *E. coli* mutants, confirming the conserved function [85]. However, the failure to methylate the conserved adenosines in the respective rRNA seems to have a different impact in various organisms: mutations of KsgA in *E. coli* led to mild reduction of translation rates and growth, while disruption of DIM1 in yeast is lethal [85]. The reason for that discrepancy might be the fact that in contrast to *E. coli* KsgA, DIM1 is additionally required for the pre-rRNA processing at cleavage sites A1 and A2 of the 18S rRNA [86].

The plant homolog of KsgA, Paleface1 (PFC1), was identified in a screen for chilling-sensitive Arabidopsis mutants [50]. The *pfc1* mutant and RNAi lines showed chlorosis and impaired chloroplast development when exposed to cold (5°C) but were indistinguishable from the WT when grown at 22°C. Sequence analysis revealed that PFC1 indeed encodes a conserved rRNA methylase required for rRNA adenine demethylation (Figure 2). Primer extension assays showed that in *pfc1* the two adenosines at the 3’ end of the chloroplast 16S rRNA were not methylated. The question whether PFC1, like DIM1, is involved in 16S rRNA processing was not addressed in this study.

To better understand the yet elusive mechanisms of m^6^A methylation in plants in general and in chloroplasts in particular, more in-depth studies are needed. The photoautotrophically viable *pfc1* mutant seems to be a good candidate for exploring whether m^6^A dimethylases, such as PFC1, act alone or in a complex together with other factors and whether the methylation is reversible. Last but not least, the question how PFC1 as an rRNA adenine dimethyltransferase (rAD) mediates chilling-resistance appears an exciting topic and needs to be addressed in the future (Figure 2).

### 5.4. m^2^G Modification of the Plastid 16S rRNA

The m^2^G methylation is the most common mark of the 16S rRNA in *E. coli* involving at least 10 enzymes and generally occurs in the latest stages of 30S assembly [87]. For example, the 16S rRNA methyltransferase RsmD installs the m^2^G methylation of this RNA at position 966 in *E. coli* [88]. Presumably as an elevated inhibitory effect on translation in the cold, the absence of RsmD and RsmB or only RsmJ responsible for the m^2^G1516 methylation of the 16S rRNA increased sensitivity at low temperature in *E. coli* [89]. Structural analysis of the 16S rRNA revealed that the G966 position corresponds to the G915 position of the chloroplast 16S rRNA in Arabidopsis [90]. Accordingly, the orthologous chloroplast RsmD protein in Arabidopsis is responsible for m^2^G methylation at position 915 as revealed by poisoned primer extension analysis. Similar to many other mutants affected in plastid ribosome assembly, lack of the chloroplast RsmD protein has no effect on germination and growth under normal conditions or under several abiotic stress conditions such as dehydration stress, salt stress, or ABA application but affects expression of plastid proteins at low temperature leading to decreased cold stress tolerance of Arabidopsis. Further analyses are needed to determine whether several described factors important for ribosome assembly indeed primarily evolved to confer cold resistance or whether cold sensitivity is prevalent simply because of decreased translational efficiency.

### 5.5. RNA Ribose Methylation

Among many other RNA species eukaryotic rRNAs and snRNAs are known to be highly modified by abundant 2′-*O*-ribose methylation (Nm), but the precise molecular function of most RNA ribose methylations is still not understood [91]. It has been assumed that Nm impacts RNA structure, stability, and interactions. Recent data suggest that Nm preferentially stabilizes alternative secondary structures, which involves pairing of Nm-modified nucleotides [92]. By applying the high-throughput sequencing method RiboMeth-seq, five almost identical Nm sited have been identified in chloroplast and mitochondrial rRNAs suggesting that the same or related bacterial-derived, stand-alone protein enzymes perform the methylation in both organelles [93]. The position of methylation sites suggests that they are relevant for the joining of the large and small ribosomal subunits and the function of the peptidyl transferase center. However, further analyses are needed to pinpoint the primary function of this modification in plant organelles.

## 6. Polyadenylation

In contrast to the mostly stabilizing role of poly(A) tails at the 3’ ends of cytoplasmic RNAs in eukaryotes, polyadenylation in eubacteria, chloroplasts, and mitochondria as well as in nuclei of diverse eukaryotes promotes the rapid exonucleolytic degradation of RNAs [94]. Several enzymes such as nucleotidyltransferase (CCA-NTR), poly(A) polymerase I (PAP I), which produce homopolymeric poly(A)-tails and is lacking in *Bacillus subtilis* and polynucleotide phosphorylase (PNPase) were shown to be responsible for RNA 3’-tail synthesis in bacteria. Besides the potential to add A, G, C, and U residues to an RNA chain, PNPases are also known for their processive 3’–5’ degradation activity. The RNA chaperone Hfq stimulates initiated polyadenylation, thereby increasing the processivity of PAP. RNA degradation involves a large set of exo- and endonucleases as well as the action of the eubacterial degradosome [94].

In cyanobacteria and chloroplasts polyadenylation and exonucleolytic degradation is performed by PNPase and no enzymatically active PAP I homolog has been identified [1]. Knockdowns and knockouts of the chloroplast PNPase resulted in a distinct increase in homopolymeric polyadenylated RNAs indicating the presence of a less active PAP and/or NTR within this organelle [95,96]. In accordance with this, the enzymatically active nucleotidyltransferase NTR3 localizes to both chloroplasts and mitochondria of Arabidopsis [97]. Due to the prevalence of PNPase activity, the posttranscriptionally extended chloroplast 3’ ends mostly contain heteropolymeric poly(A)-rich sequences of up to several hundred nucleotides in spinach as revealed by sequencing of PCR product using oligo dT-primed cDNAs [98,99,100]. However, the frequency of homo- and heteropolymeric tails may differ between species [101].

RNA polyadenylation in chloroplasts is presumably not the initial step in regulation of RNA degradation. Endonucleolytic degradation of plastid RNAs is performed by numerous RNases including RNase J, CSP41a/b, and a degradosome-like complex consisting of RNase E, the rho-containing RHON1 protein, helicases, and additional components [2,3,6,102].

The majority of 3’ tail addition sites coincided with endonucleolytic cleavage sites indicating that RNA degradation and intron removal is initiated by endonucleolytic activities that create substrates for polyadenylation and that stable stem-loop structures may prevent not only degradation but also polyadenylation [6,96,99]. On the other hand, polyadenylated stretches serve as platforms for processive exonucleases, such as PNPase, RNase II/R, and/or other proteins supporting unwinding of stable structures and subsequent degradation [8]. It is important to mention that to this day it is unclear whether there is also a polyadenylation-independent pathway for RNA degradation in chloroplasts [99].

Although we already know so much about the mechanisms of plastid RNA degradation and the factors involved, the role and regulation by environmental factors and endogenous influences is virtually unknown. Similarly, nothing is known about the time course of the initial regulation of RNA stability potentially driven by environmental and endogenous factors that could modulate endonuclease activity and/or polyadenylation and thus regulate RNA half-life within chloroplasts. In this context it is worth noting that downregulation of the chloroplast PNPase had only minor effects on mRNA levels, implying that other mechanisms than polyadenylation are key players in the regulation of mRNA steady-state levels [95]. An indication of regulation may be provided by the cold sensitivity of a PNPase-less mutant in *Escherichia coli* [103].

## 7. Polyuridylation

In contrast to housekeeping transcripts, mostly mRNAs in the fucoxanthin dinoflagellate are edited and/or contain 3’-poly(U) tail extensions. Both posttranscriptional modifications were acquired following serial endosymbiotic events. Many of the characteristics were also found in peridin dinoflagellates whose plastomes consist of many mini-circles encoding one or few photosynthetic proteins. However, other dinoflagellates are lacking both modifications, demonstrating that polyuridylation was acquired by a common ancestor in the fucoxanthin dinoflagellate lineage [104]. Data suggest that poly(U) additions are highly sequence specific and that polyuridylation occurs in the 3’ end of mRNAs, whereas tRNAs and plastid RNAs in apicomplexans that lost photosynthesis lack polyuridylation [105]. Although the function of polyuridylation in alveolates remains unclear, it has been proposed to be important for discriminating functional mRNAs from pseudogene-derived RNAs [104]. Polyuridylation of some RNAs may also be associated with editing as has been described for mitochondria in trypanosomes [106].

In a recent study poly(U) tails were also found in plastid RNAs within the Cladophorales green alga lineage [107]. Thus, polyuridylation may have convergently evolved in different photosynthetic lineages. It has been hypothesized that poly(U) extensions of 3’ processed transcripts in this green algal order may be related to the highly reduced size of the chloroplast genome consisting of linear single-stranded DNAs that fold into hairpin structures rather than marking them for degradation [107].

With the noticeable exception of budding yeast, uridylation at different nuclear and cytoplasmic RNA populations is catalyzed by terminal uridylyl transferases or poly(U) polymerases in eukaryotes where it is associated in a positive or negative way with RNA degradation, maturation of microRNAs, and the maintenance of U6 small nuclear RNAs [108]. With the exception of Cladophorales poly(U) tails have never been observed in chloroplasts of the green lineage but at the 3’ ends of truncated transcripts in mitochondria of Chlamydomonas indicating an association with RNA degradation in this organelle [97].

Elucidating the factors, targets, and possible interacting partners involved in plant organelle polyuridylation, its temporal and spatial regulation and its implication in regulation of gene expression under changing conditions will be a major task as virtually nothing is known about it [109].

## 8. Pseudouridylation

Pseudouridine (ribosyluracil; Ψ) is a naturally generated modified nucleoside that plays a central role in all kingdoms of life. Most studies performed preferentially in mammals, yeast, and bacteria demonstrated an important function of post-transcriptional pseudouridylation in stabilizing RNA secondary structures that affect RNA localization, ribosome assembly as well as efficiency, and fidelity of protein biosynthesis to regulate growth, development, and responses to various stresses [110].

So far, little is known about the function and components involved in the Ψ modifications in plant cytoplasmic and organellar RNAs [111,112]. Ψ consists of β-D-ribofuranose with a covalently bound C1 to the C5 of the base uracil instead of N1 (Figure 1). This modification thermodynamically stabilizes the base-pairing properties of pseudouridines toward adenosines compared to the U-A base pair due to the additional hydrogen bond formed by N1.

Pseudouridine is the most abundant nucleoside modification across all RNA species amounting up to 0.4% of all uridines and is prevalently found in partially conserved positions in rRNAs, snRNAs, and the TΨC-loop (Ψ55), the D stem and the anticodon stem and loop of tRNAs but generally to a lesser extent in mRNAs [17,110,111]. Thus, it is often termed as “the fifth nucleoside”. Interestingly, changes in Ψ modifications in mRNAs are induced by heat stress, oxidative stress, diseases, cancer, and nutrient deficiency pointing to an important function of Ψ in development, stress response, and acclimation to environment cues [113]. A genome-wide pseudouridine-sequencing approach in Arabidopsis revealed pseudouridylation in 1.21% of all detected mRNAs with an enrichment in the first nucleotide of triple codons and to a lesser extent in the 5’ and 3’ UTRs [112]. In accordance with the proposed function enriched pseudouridylated mRNAs encode components involved in responses to environmental stimuli and stress, metabolic and biosynthetic processes, as well as energy generation and photosynthesis [112].

Ψ writers are synthases (PUS enzymes) that use aspartate as the nucleophile. Based on their amino acid sequences, Ψ synthases were grouped into the five families RluA, RsuA, TruA, TruB and TruD, which all modify specific, non-overlapping targets [114]. They catalyze post-transcriptionally the isomerization of uridine to generate Ψ. Depending on the enzymes, these modifications require consensus sequences or structural properties. PUS enzymes either act alone in the recognition of the substrate and the isomerization of uridine to pseudouridine in an RNA-independent manner or they form RNA-guided complexes with ribonucleoproteins and additional four common core proteins. The guide RNA is responsible for recognition and pseudouridylation at sequence-specific sites [115]. Several methods for the detection of Ψ are based on chromatography or on the specific binding of carbodiimide to the modified site preventing reverse transcription.

Although the functions of most Ψs are still enigmatic, it is generally believed that they primarily serve a structural role in the respective RNAs. Besides profound phenotypic effects, Ψ mutants often show no obvious phenotypic deviations from the WT, implying also regulatory roles of Ψ. Interestingly, Ψ was found to undergo dynamic changes in response to heat and to affect RNA levels in yeast [116]. Moreover, point mutations in the yeast Ψ synthase Cbf5p show cold- and heat-sensitive growth phenotypes and reduced Ψ content in rRNAs [110].

Despite increasing knowledge, still little is known about the precise mechanisms and functions of RNA pseudouridylation and proteins involved in plants. Out of 21 putative pseudouridine synthases encoded in the Arabidopsis genome, 8 were annotated as chloroplast enzymes, indicating an important function of Ψ in this organelle (www.arabidopsis.org, accessed on 23 July 2021).

First evidence for pseudouridylation of tRNAs in chloroplasts came from in vitro experiments [117]. A chloroplast extract was enzymatically active in vitro at 25°C but not at 37°C, suggesting that either the Ψ synthetases are temperature-sensitive or that other temperature sensitive base modifications are a prerequisite for pseudouridylation. In any case, temperature appears to be an important factor when it comes to pseudouridylation within chloroplasts. Consistent with this, a mutant of the chloroplast Ψ synthase TCD3 in rice displayed thermo-sensitive changes in leaf color under cold stress and was not viable at 20°C, but showed normal growth at 32°C, indicating an important role of Ψ at low temperature [118]. The thermo-sensitivity of the *tcd3* mutant is reflected by the differential expression pattern of plastid and presumably as a feedback effect also of cytoplasmic transcripts for chlorophyll biosynthesis, photosynthesis, plastid gene expression, and chloroplast development at 20°C and a complete restoration to WT levels at 32°C. In accordance with this, TCD3 is upregulated in the cold, indicating an important response at lower temperatures [118].

The chloroplast Ψ synthase SVR1 (At2g39140) fulfills all the sequence criteria of a bona fide Ψ synthase and was identified as a suppressor of the *var2*-mediated leaf variegation in Arabidopsis [119]. Eleven out of 15 Ψ sites were missing in rRNA species in the *svr1* mutants that displayed an impaired rRNA processing and/or ribosomal assembly accompanied by decreased rates of chloroplast translation and reduced accumulation of chloroplast proteins [112]. Thus, the consequences of loss of SRV1 emphasize the role of Ψ in ribosome assembly and chloroplast translation. The presence of 4 unaffected Ψ in rRNAs in *svr1* mutants indicates the existence of additional Ψ synthases in the chloroplast. The deficiencies found in the *svr1* likely decreased the demand for VAR2 activity during chloroplast biogenesis and thus, suppressed the *var2* phenotype [119]. Although it seems likely, it still remains unclear whether an enzymatically active Ψ synthase is required for rRNA processing and/or *var2* suppression. In another study it was shown that mutations of *SVR1* reduce plant sensitivity to Pi starvation [120]. TCD3 in rice belongs to the RsuA family and shows 60.6% sequence similarity to SVR1 in Arabidopsis, implying that TCD3 possibly represents an enzymatically active Ψ synthase.

The chloroplast protein MAA2 in Chlamydomonas is related to Ψ synthases and has been recruited for the assistance of a Ψ-independent group II intron trans-splicing by an unknown mechanism. However, it remains unclear whether the enzyme function has been maintained within the organelle [121].

## 9. RNA Editing

The translation of genetic information from DNA to protein requires an intermediate product, the RNA, that generally follows the DNA sequence. However, in some cases the sequence of the RNA does not match the sequence of the cognate DNA. The post-transcriptional processing step, in which RNA bases are altered relatively to the corresponding DNA sequence is known as RNA editing [122]. This process is pervasive in chloroplasts and mitochondria and consists almost exclusively of C-U substitutions. Here we briefly summarize the important findings that have arisen from more than three decades of research mainly concerning the significance, the factors involved, and the mechanisms underlying plastid RNA editing. For more details on organellar RNA editing, we refer to recent and extensive review articles [123,124,125].

RNA editing was first discovered in 1989 in mitochondria [126,127,128]. Two years later, it was reported to occur also in chloroplasts [129]. Advancing sequencing approaches allowed later on genome-wide editing analyses and revealed that mitochondrial transcripts of Arabidopsis contain several hundred editing sites [130], while the number of 34 editing events in plastid RNAs is comparably low [125]. The fact that RNA editing is widely spread in land plant organelles but has not been observed in bacteria or algae along with the circumstance that most of the edited RNA bases in organelles restore codons, which are highly conserved in their cyanobacterial ancestor, supports the widely held view that plant RNA editing evolved to correct DNA mutations presumably caused by the increased exposure to mutagenizing UV rays that plants faced during the transition from an aquatic to terrestrial way of life [131]. Besides the role in debugging the genetic code, plastid RNA editing has been proposed to have an additional regulatory function. This notion arose, inter alia, from the observation that several partially edited sites are differentially edited in various plant tissues or under changing growth conditions and upon external stimuli implying a function in development and acclimation processes [132,133,134,135].

**Figure 3:**
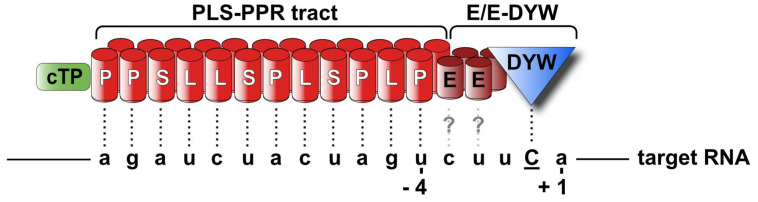
Mechanism of plastid RNA editing by a DYW-containing PLS-PPR protein. A PPR protein composed of a chloroplast target sequence (cTP), 12 tandemly arranged PLS repeats (double long barrels represent the two alpha helices of each repeat), the E/E extension (double short barrels), and a DYW domain (triangle) is shown. The PPR protein recognizes the target RNA via the PLS tract in a one-repeat-to-one-nucleotide manner with its last repeat bound to the nucleotide -4, positioning the catalytic DYW domain to the C residue to be edited. The possible involvement of the E domains in nucleotide binding is illustrated by question marks.

First steps towards the understanding of the determinants for plastid RNA editing came from a transplastomic approach in tobacco plants expressing chimeric *ndhB* transcript variants. 5’ and 3’ deletions or nucleotide substitutions adjacent to editing sites within *ndhB* transcripts provided evidence that *cis*-acting elements located in the -12/-2 region upstream of the editing site are essential for RNA editing [136]. About one decade later a pioneer study reported about the first *trans*-acting factor required for plastid RNA editing, the pentatricopeptide repeat protein (PPR) CRR4 [137]. The CRR4 protein binds a sequence located upstream of the editing site [138] and facilitates the conversion from ACG to AUG, creating the correct *ndhD* initial codon. Since then, numerous other editing mutants and factors have been described revealing that PPR proteins are key players and the specificity factors for organellar RNA editing (characterized plastid PPR editing factors are summarized in Supplementary Table S2) (Figure 3).

The PPR protein family was first described in Arabidopsis and consists of about 450 members [139]. PPRs are nucleus-encoded, organellar-specific RNA-binding proteins defined by a tandem array of degenerate 35 amino acid repeats that form helix-loop-helix structures [10,140]. The PPR motifs are arranged consecutively and form a right-handed super helix that binds RNA in a sequence-specific, contiguous 1-motif to 1-nucleotide manner [141]. Extensive experimental analyses of PPR proteins and their targets—especially the elucidation of the structure of PPR proteins bound to RNA [141]—have revealed that the RNA base recognition is mainly determined by the amino acid motif at positions 5 and 35 of each motif. Based on these findings, the so-called RNA recognition code has been developed and experimentally confirmed [142,143]. According to the length of the repeats and the presence of additional C-terminal domains, PPRs are split into two sub-classes: P- and PLS-class PPR proteins. P-class PPRs exclusively contain canonical repeats of 35 aa and are involved in all RNA metabolism processes such as transcription, translation, stabilization, editing, processing, and splicing (reviewed in [144]). PPR proteins of the PLS-subfamily are dedicated to RNA editing, as all PPR proteins involved in RNA editing belong to this subgroup. They contain long (L) and short (S) repeats beside the “classical” (P) 35 aa repeat. PLS-class PPR proteins are plant-specific and usually harbor an additional C-terminal domain: the “E” (extended) domain and the “DYW” domain named after the last three amino acids.

However, what is the mechanism of RNA editing? It is well understood that the RNA to be edited is specifically recognized and bound by the PLS tract of the PLS-class PPR protein singling out the correct target mRNA. The PLS-PPR protein binds the RNA target in a way that its last PPR motif is associated with the fourth nucleotide upstream of the editable C residue. The PLS tract is followed by the E domains that show similarities to the PPR repeats and are thought to either engage in protein-protein interaction or to contribute to RNA base recognition [145], however their function is not yet entirely understood. A special attention has been paid to the DYW domain that bears a motif similar to the conserved cytidine/deoxycytidylate deaminase motif (C/HXE(X)nPCXXC, InterPro: IPR002125) [125] and has been therefore proposed to be the catalytic domain for organellar RNA editing. The first direct evidence for the editase activity of the DYW domain was provided in a recent study carried out heterologously in *E. coli*, where E/E-DYW-containing PLS-PPR proteins from *Physcomitrella patens* were shown to be able to perform efficient and targeted C-to-U editing in vivo [146]. Shortly after, the catalytic activity of the DYW domain was additionally confirmed in in vitro experiments with purified proteins, demonstrating that DYW-PPR proteins alone are sufficient for C-to-U editing [147]. A model of target recognition and editing by a DYW-PPR protein is shown in Figure 3.

Although *Physcomitrella patens* organellar editing seems not to require the interplay of factors other than DYW-PPR proteins, mutant analyses in angiosperms suggest that the editing machinery of higher plants is more complex and relies on additional *trans*-acting proteins. For example, the previously mentioned factor CRR4 is a truncated PLS-PPR protein that lacks the DYW domain and thus belongs to the so-called E-class proteins. To be able to catalyze the C deamination of the *ndhD*-1 editing site, CRR4 requires the interaction with DYW1, a small DYW domain-containing protein missing a PPR tract [148]. Two other proteins, NUWA and DYW2, that belong to the P-type PPR and to the “small” PPR-DYW protein class, respectively, are dually targeted to mitochondria and chloroplasts and seem to be core members of E-type PPR editosomes, as many of the defective editing sites in the embryo lethal *nuwa* and *dyw2* mutants are targets of E-type PPR proteins [149]. It appears that E-type PPR proteins recruit factors such as DYW1 and DYW2 to compensate for their missing DYW domain.

Three other protein classes, that in contrast to PPR proteins act rather unspecifically, were identified as essential components of the plant organellar editosomes: The Multiple Organellar RNA editing Factors (MORFs) [150], proteins containing RNA Recognition Motifs such as ORRM and CP31 [151,152] and OZ proteins [153]. Among those best characterized are the MORF proteins. MORF2 and MORF9 localize to chloroplasts and participate in nearly all editing events, while MORF5 and MORF8 are dual targeted to plastids and mitochondria [150]. MORF proteins also seem to facilitate the binding of PPR proteins to their target RNAs, as it was shown that the interaction of MORF9 with PLS-PPR proteins induces significant conformational changes of the PLS tract, which in turn increase the RNA binding affinity [154].

It also remains unknown how PPR proteins form a functional editosome and whether associated non-PPR proteins, such as RNA chaperones, are generally involved in unwinding stable RNA structures to allow access to the targets or to fulfill regulatory functions [2,155].

Taken together, increasing mutagenic environmental conditions forced the acquisition of PPR proteins with editase activity in order to reverse the DNA mutations in plant organelles. Many other non-PPR factors co-evolved to support organellar editing of flowering plants, but their functions are still under investigation.

## Data Availability

Not applicable.

## Supplementary Materials

The following are available online at https://www.mdpi.com/article/10.3390/genes12081121/s1, Table S1: Summary of chloroplast- and nucleus-encoded genes found in m^6^A epitrancriptomes of different accessions of Arabidopsis. The information of this table was extracted from 3 published epitranscriptome data and the ‘m^6^A-Atlas’ tool was used to predict updated numbers of methylation sites (www.xjtlu.edu.cn/biologicalsciences/atlas) [1]., Table S2: Summary of characterized PPR proteins involved in organellar RNA editing in Arabidopsis. The information of this table was extracted from the Prepact database (http://www.prepact.de/prepact-main.php).
