# Supplementary material for: The Chloroplast Epitranscriptome: Factors, Sites, Regulation, and Detection Methods
Source: Genes (Basel). 2021 Jul 24;12(8):1121. doi: 10.3390/genes12081121 (PMC8394491; doi:10.3390/genes12081121)
Supplement: Supplementary file 1 [file genes-12-01121-s001.zip › genes-1290778-supplementary.pdf]

Table S1. Summary of chloroplast- and nucleus-encoded genes found in m^{6}A epitrancriptomes of different accessions of *Arabidopsis*. The information of this table was extracted from 3 published epitranscriptome data and the 'm^{6}A-Atlas' tool was used to predict updated numbers of methylation sites (www.xjtlu.edu.cn/biologicalsciences/atlas) [1].

Gene(s)	Name(s)	Gene Ontology	Accession(s)	m^{6}A-Atlas	Reference(s)
AT1G24490	ALB4	Plastid organization	Con-0	1	[2] Supplementary Data 5
AT5G45390	CLPP4	Plastid organization	Con-0	4	
AT3G19720	ARC5	Plastid organization	Con-0	3	
AT3G48870	HSP93-III	Plastid organization	Con-0	2	
AT2G48120	PAC	Plastid organization	Con-0	0	
AT1G24490	ALB4	Plastid organization	Hen-16	1	
AT5G10490	MSL2	Plastid organization	Hen-16	0	
AT3G17040	HCF107	Plastid organization	Hen-16	4	
AT1G63900	DAL1	Plastid organization	Hen-16	5	
AT5G02250	EMB2730	Plastid organization	Hen-16	3	
AT3G16140	PSAH-1	photosynthesis	Con-0	0	
AT1G70760	CRR23	photosynthesis	Con-0	1	
AT4G28660	PSB28	photosynthesis	Con-0	0	
AT1G06680	PSBP-1	photosynthesis	Con-0	1	
AT3G61470	LHCA2	photosynthesis	Con-0	1	
AT5G66570	PSBO1	photosynthesis	Con-0	7	
AT4G28750	PSAE-1	photosynthesis	Con-0	5	
AT5G16440	IPP1	photosynthesis	Con-0	6	
AT3G16250	NDF4	photosynthesis	Con-0	9	
AT2G05100	LHCB2.1	photosynthesis	Con-0	1	
AT1G32060	PRK	photosynthesis	Con-0	4	
AT1G55670	PSAG	photosynthesis	Col-0	0	

AT2G05100	LHCB2	photosynthesis	Col-0	1	[3] Table 6
AT3G08940	LHCB4	photosynthesis	Col-0	4	
AT3G21055	PSBTN	photosynthesis	Col-0	0	
AT1G67740	PSBY	photosynthesis	Col-0	5	
AT1G03130	PSAD-2	photosynthesis	Col-0	12	
AT2G34420	LHB1B2	photosynthesis	Col-0	1	
AT1G15820	LHCB6	photosynthesis	Col-0	0	
AT4G10340	LHCB5	photosynthesis	Col-0	5	
AT1G61520	LHCA3	photosynthesis	Col-0	3	
AT2G06520	PSBX	photosynthesis	Col-0	0	
AT3G15190	PRPS20	photosynthesis	Col-0	0	
ATCG01160	RRN5S	Chloroplast rRNA	Col-0	0	[4] Table 6
ATCG01210	RRN16S	Chloroplast rRNA	Col-0	0	
ATCG00950	RNA23S	Chloroplast rRNA	Col-0	5	
ATCG00920	RRN16S	Chloroplast rRNA	Col-0	0	
ATCG00660	RPL20	ribosomal proteins	Col-0	0	
ATCG00650	RPS18	ribosomal proteins	Col-0	0	
ATCG00400	tRNA-Leu	tRNA	Col-0	2	
ATCG00550	PSBJ	photosystem reaction proteins	Col-0	0	
ATCG00510	PSAI	photosystem reaction proteins	Col-0	0	
ATCG00140	ATPH	ATP synthase subunit C	Col-0	0	
ATCG01130	TIC214	Ycf1 protein	Col-0	5	
ATCG01010	NDHF	NADH-Ubiquinone oxidoreductase	Col-0	3	
ATCG01040	YCF5	Cytochrome C assembly protein	Col-0	0	

References:

1. Tang, Y.; Chen, K.; Song, B.; Ma, J.; Wu, X.; Xu, Q.; Wei, Z.; Su, J.; Liu, G.; Rong, R.; et al. m6A-Atlas: a comprehensive knowledgebase for unraveling the N6-methyladenosine (m6A) epitranscriptome. *Nucleic Acids Res.* **2021**, *49*, D134–D143, doi:10.1093/nar/gkaa692.
2. Luo, G.Z.; Macqueen, A.; Zheng, G.; Duan, H.; Dore, L.C.; Lu, Z.; Liu, J.; Chen, K.; Jia, G.; Bergelson, J.; et al. Unique features of the m6A methylome in Arabidopsis thaliana. *Nat. Commun.* **2014**, *5*, 1–8, doi:10.1038/ncomms6630.
3. Wan, Y.; Tang, K.; Zhang, D.; Xie, S.; Zhu, X.; Wang, Z.; Lang, Z. Transcriptome-wide high-throughput deep m6A-seq reveals unique differential m6A methylation patterns between three organs in Arabidopsis thaliana. *Genome Biol.* **2015**, *16*, 1–26, doi:10.1186/s13059-015-0839-2.
4. Wang, Z.; Tang, K.; Zhang, D.; Wan, Y.; Wen, Y.; Lu, Q.; Wang, L. High-throughput m6A-seq reveals RNA m6A methylation patterns in the chloroplast and mitochondria transcriptomes of Arabidopsis thaliana. *PLoS One* **2017**, *12*, e0185612, doi:10.1371/journal.pone.0185612.

Table S2. Summary of characterized PPR proteins involved in organellar RNA editing in Arabidopsis. The information of this table was extracted from the Prepact database (http://www.prepact.de/prepact-main.php).

Abbreviation	Name	GenPept	Organism	PLS repeats	Terminal domain	Reference(s)	Editing sites
CRR4	Chlororespiratory Protein 4	NP_182060.2	*Arabidopsis thaliana*	14	dDYW	[1] [2] [3] [4] [5] [6] [7] [8]	ndhDeU2TM (cp)
AEF1	atpF Editing Factor 1	NP_188854.1	*Arabidopsis thaliana*	18	dDYW	[9]	atpFeU92PL (cp) nad5eU1580SL (mt)
CLB19 PDE247	Chloroplast Biogenesis 19 Pigment Defective 247	NP_172066.3	*Arabidopsis thaliana*	10	dDYW	[10] [5] [7] [11] [12] [13] [14]	clpPeU559HY (cp) rpoAeU200SF (cp)
CRR21	Chlororespiratory Protein 21	NP_200385.1	*Arabidopsis thaliana*	20	dDYW	[3] [5] [7] [15]	ndhDeU383SL (cp)
CRR22	Chlororespiratory Protein 22	NP_172596.1	*Arabidopsis thaliana*	16	DYW	[16]	ndhBeU746SF (cp)

						[5]<br>[17]	ndhDeU887PL (cp)<br>rpoBeU551SL (cp)
ECB2<br>VAC1	Early Chloroplast Biogenesis 2<br>Vanilla Cream 1	NP_173004.1	*Arabidopsis thaliana*	17	dDYW	[18]<br>[19]<br>[20]	accDeU+101 (cp)<br>accDeU794SL (cp)<br>ndhFeU290SL (cp)<br>ndhGeU50SF (cp)<br>petLeU5PL (cp)<br>rpoAeU200SF (cp)<br>rpoC1eU488SL (cp)
CRR28	Chlororespiratory Protein 28	NP_176180.1	*Arabidopsis thaliana*	10	DYW	[16]<br>[5]<br>[15]<br>[21]<br>[14]	ndhBeU467PL (cp)<br>ndhDeU878SL (cp)
DOT4<br>FLV	Defectively Organized Tributaries 4<br>Flavodentata	NP_193610.1	*Arabidopsis thaliana*	18	DYW	[22]	rpoC1eU488SL (cp)
ELI1	Editing Lacking Insertional 1	NP_195454.1	*Arabidopsis thaliana*	10	DYW	[22]	ndhBeU830SL (cp)
LPA66	Low PSII Accumulation 66	NP_199702.1	*Arabidopsis thaliana*	11	DYW	[23]	psbFeU77SF (cp)
OTP80	Organelle Transcript Processing 80	NP_200728.2	*Arabidopsis thaliana*	11	dDYW	[16]<br>[15]	rpl23eU89SL (cp)
OTP81<br>QED1	Organelle Transcript Processing 81<br>Quintuple Editing Factor	NP_180537.1	*Arabidopsis thaliana*	14	DYW	[16]<br>[24]	accDeU+101 (cp)<br>matKeU640HY (cp)<br>ndhBeU872SL (cp)<br>rpoBeU2432SL (cp)<br>rps12i114eU58 (cp)
OTP82	Organelle Transcript Processing 82	NP_172286.1	*Arabidopsis thaliana*	13	DYW	[25]<br>[17]	ndhBeU836SL (cp)<br>ndhGeU50SF (cp)

						[7]<br>[11]	
OTP84	Organelle Transcript Processing 84	NP_191302.2	*Arabidopsis thaliana*	17	DYW	[16]<br>[26]	ndhBeU1481PL (cp)<br>ndhFeU290SL (cp)<br>psbZeU50SL (cp)
OTP85	Organelle Transcript Processing 85	NP_178398.1	*Arabidopsis thaliana*	10	DYW	[16]<br>[15]	ndhDeU674SL (cp)
OTP86	Organelle Transcript Processing 86	NP_191896.2	*Arabidopsis thaliana*	19	DYW	[16]	rps14eU80SL (cp)
PpPPR_45	Pentatricopeptide Repeat Protein 45	XP_024389794.1	*Physcomitrella patens*	25	DYW	[27]	rps14eU-1 (cp)<br>rps14eU2TM (cp)
RARE1	Required for accD RNA Editing 1	NP_196831.1	*Arabidopsis thaliana*	15	DYW	[5]<br>[24]<br>[21]<br>[14]	accDeU794SL (cp)
YS1	Yellow Seedlings 1	NP_001189950.1	*Arabidopsis thaliana*	16	DYW	[28]<br>[5]	rpoBeU338SF (cp)
CREF7	Chloroplast RNA Editing Factor 7	NP_201453.1	*Arabidopsis thaliana*	10	DYW	[29]<br>[26]	ndhBeU1255HY (cp)
CREF3	Chloroplast RNA Editing Factor 3	NP_188050.1	*Arabidopsis thaliana*	10	DYW	[29]	psbEeU214PS (cp)
PDM1<br>SEL1	Pigment Deficient Mutant 1<br>Seedling Lethal 1	NP_193587.4	*Arabidopsis thaliana*	14	dE	[30]<br>[31]	accDeU+101 (cp)<br>accDeU794SL (cp)
PPR6	Pentatricopeptide Repeat Protein 6	XP_015639788.1	*Oryza sativa Japonica Group*	17	dDYW	[32]	ndhBeU737PL (cp)
PGL1	Pale Green Leaves 1	XP_015618645.1	*Oryza sativa Japonica Group*	13	DYW	[33]	ccmFCeU543VV (mt)<br>ndhDeU878SL (cp)
ECD1<br>EMB2261	Early Chloroplast Development 1<br>Embryo-defective 2261	NP_190486.2	*Arabidopsis thaliana*	17	DYW	[34]<br>[35]	accDeU794SL (cp)<br>ndhBeU836SL (cp)<br>ndhDeU878SL (cp)

	ndhFeU290SL (cp)<br>petLeU5PL (cp)<br>rps14eU149PL (cp)

References

1. Kotera, E.; Tasaka, M.; Shikanai, T. A pentatricopeptide repeat protein is essential for RNA editing in chloroplasts. *Nature* **2005**, *433*, 326–330, doi:10.1038/nature03229.
2. Okuda, K.; Nakamura, T.; Sugita, M.; Shimizu, T.; Shikanai, T. A pentatricopeptide repeat protein is a site recognition factor in chloroplast RNA editing. *J. Biol. Chem.* **2006**, *281*, 37661–37667, doi:10.1074/jbc.M608184200.
3. Okuda, K.; Myouga, F.; Motohashi, R.; Shinozaki, K.; Shikanai, T. Conserved domain structure of pentatricopeptide repeat proteins involved in chloroplast RNA editing. *Proc. Natl. Acad. Sci. U. S. A.* **2007**, *104*, 8178–8183, doi:10.1073/pnas.0700865104.
4. Okuda, K.; Habata, Y.; Kobayashi, Y.; Shikanai, T. Amino acid sequence variations in Nicotiana CRR4 orthologs determine the species-specific efficiency of RNA editing in plastids. *Nucleic Acids Res.* **2008**, *36*, 6155–6164, doi:10.1093/nar/gkn629.
5. Robbins, J.C.; Heller, W.P.; Hanson, M.R. A comparative genomics approach identifies a PPR-DYW protein that is essential for C-to-U editing of the Arabidopsis chloroplast accD transcript. *RNA* **2009**, *15*, 1142–1153, doi:10.1261/rna.1533909.
6. Chateigner-Boutin, A.L.; Des Francs-Small, C.C.; Delannoy, E.; Kahlau, S.; Tanz, S.K.; De Longevialle, A.F.; Fujii, S.; Small, I. OTP70 is a pentatricopeptide repeat protein of the e subgroup involved in splicing of the plastid transcript rpoC1. *Plant J.* **2011**, *65*, 532–542, doi:10.1111/j.1365-313X.2010.04441.x.
7. Hayes, M.L.; Giang, K.; Mulligan, R.M. Molecular evolution of pentatricopeptide repeat genes reveals truncation in species lacking an editing target and structural domains under distinct selective pressures. *BMC Evol. Biol.* **2012**, *12*, doi:10.1186/1471-2148-12-66.
8. Boussardon, C.; Salone, V.; Avon, A.; Berthomé, R.; Hammani, K.; Okuda, K.; Shikanai, T.; Small, I.; Lurina, C. Two interacting proteins are necessary for the editing of the ndhD-1 site in Arabidopsis plastids. *Plant Cell* **2012**, *24*, 3684–3694, doi:10.1105/tpc.112.099507.
9. Yap, A.; Kindgren, P.; Colas Des Francs-Small, C.; Kazama, T.; Tanz, S.K.; Toriyama, K.; Small, I. AEF1/MPR25 is implicated in RNA editing of plastid atpF and mitochondrial nad5, and also promotes atpF splicing in Arabidopsis and rice. *Plant J.* **2015**, *81*, 661–669, doi:10.1111/tpj.12756.
10. Chateigner-Boutin, A.L.; Ramos-Vega, M.; Guevara-García, A.; Andrés, C.; Gutiérrez-Nava, M.D.L.L.; Cantero, A.; Delannoy, E.; Jiménez, L.F.; Lurin, C.; Small, I.; et al. CLB19, a pentatricopeptide repeat protein required for editing of rpoA and clpP chloroplast transcripts. *Plant J.* **2008**, *56*, 590–602, doi:10.1111/j.1365-313X.2008.03634.x.
11. Kindgren, P.; Yap, A.; Bond, C.S.; Small, I. Predictable alteration of sequence recognition by RNA editing factors from arabidopsis. *Plant Cell* **2015**, *27*, 403–416, doi:10.1105/tpc.114.134189.
12. Ramos-Vega, M.; Guevara-García, A.; Llamas, E.; Sánchez-León, N.; Olmedo-Monfil, V.; Vielle-Calzada, J.P.; León, P. Functional analysis of the Arabidopsis thaliana CHLOROPLAST BIOGENESIS 19 pentatricopeptide repeat editing protein. *New Phytol.* **2015**, *208*, 430–441,

doi:10.1111/nph.13468.
13. Guillaumot, D.; Lopez-Obando, M.; Baudry, K.; Avon, A.; Rigaill, G.; Falcon De Longevialle, A.; Broche, B.; Takenaka, M.; Berthomé, R.; De Jaeger, G.; et al. Two interacting PPR proteins are major Arabidopsis editing factors in plastid and mitochondria. *Proc. Natl. Acad. Sci. U. S. A.* **2017**, *114*, 8877–8882, doi:10.1073/pnas.1705780114.
14. Hein, A.; Knoop, V. Expected and unexpected evolution of plant RNA editing factors CLB19, CRR28 and RARE1: Retention of CLB19 despite a phylogenetically deep loss of its two known editing targets in Poaceae. *BMC Evol. Biol.* **2018**, *18*, doi:10.1186/s12862-018-1203-4.
15. Okuda, K.; Shoki, H.; Arai, M.; Shikanai, T.; Small, I.; Nakamura, T. Quantitative analysis of motifs contributing to the interaction between PLS-subfamily members and their target RNA sequences in plastid RNA editing. *Plant J.* **2014**, *80*, 870–882, doi:10.1111/tpj.12687.
16. Hammani, K.; Okuda, K.; Tanz, S.K.; Chateigner-Boutin, A.L.; Shikanai, T.; Small, I. A study of new arabidopsis chloroplast rna editing mutants reveals general features of editing factors and their target sites. *Plant Cell* **2009**, *21*, 3686–3699, doi:10.1105/tpc.109.071472.
17. Okuda, K.; Shikanai, T. A pentatricopeptide repeat protein acts as a site-specificity factor at multiple RNA editing sites with unrelated cis-acting elements in plastids. *Nucleic Acids Res.* **2012**, *40*, 5052–5064, doi:10.1093/nar/gks164.
18. Yu, Q.B.; Jiang, Y.; Chong, K.; Yang, Z.N. AtECB2, a pentatricopeptide repeat protein, is required for chloroplast transcript accd rna editing and early chloroplast biogenesis in arabidopsis thaliana. *Plant J.* **2009**, *59*, 1011–1023, doi:10.1111/j.1365-313X.2009.03930.x.
19. Tseng, C.C.; Sung, T.Y.; Li, Y.C.; Hsu, S.J.; Lin, C.L.; Hsieh, M.H. Editing of accD and ndhF chloroplast transcripts is partially affected in the Arabidopsis vanilla cream1 mutant. *Plant Mol. Biol.* **2010**, *73*, 309–323, doi:10.1007/s11103-010-9616-5.
20. Cao, Z.L.; Yu, Q.B.; Sun, Y.; Lu, Y.; Cui, Y.L.; Yang, Z.N. A Point Mutation in the Pentatricopeptide repeat Motif of the AtECB2 Protein Causes Delayed Chloroplast Development. *J. Integr. Plant Biol.* **2011**, *53*, 258–269, doi:10.1111/j.1744-7909.2011.01030.x.
21. Hein, A.; Polsakiewicz, M.; Knoop, V. Frequent chloroplast RNA editing in early-branching flowering plants: Pilot studies on angiosperm-wide coexistence of editing sites and their nuclear specificity factors. *BMC Evol. Biol.* **2016**, *16*, doi:10.1186/s12862-016-0589-0.
22. Hayes, M.L.; Giang, K.; Berhane, B.; Mulligan, R.M. Identification of two pentatricopeptide repeat genes required for rna editing and zinc binding by c-terminal cytidine deaminase-like domains. *J. Biol. Chem.* **2013**, *288*, 36519–36529, doi:10.1074/jbc.M113.485755.
23. Cai, W.; Ji, D.; Peng, L.; Guo, J.; Ma, J.; Zou, M.; Lu, C.; Zhang, L. LPA66 is required for editing psbF chloroplast transcripts in Arabidopsis. *Plant Physiol.* **2009**, *150*, 1260–1271, doi:10.1104/pp.109.136812.
24. Wagoner, J.A.; Sun, T.; Lin, L.; Hanson, M.R. Cytidine deaminase motifs within the DYW domain of two pentatricopeptide repeat-containing proteins are required for site-specific chloroplast RNA editing. *J. Biol. Chem.* **2015**, *290*, 2957–2968, doi:10.1074/jbc.M114.622084.
25. Okuda, K.; Hammani, K.; Tanz, S.K.; Peng, L.; Fukao, Y.; Myouga, F.; Motohashi, R.; Shinozaki, K.; Small, I.; Shikanai, T. The pentatricopeptide repeat protein OTP82 is required for RNA editing of plastid ndhB and ndhG transcripts. *Plant J.* **2010**, *61*, 339–349, doi:10.1111/j.1365-313X.2009.04059.x.
26. Hayes, M.L.; Dang, K.N.; Diaz, M.F.; Mulligan, R.M. A conserved glutamate residue in the C-terminal deaminase domain of pentatricopeptide repeat proteins is required for RNA editing activity. *J. Biol. Chem.* **2015**, *290*, 10136–10142,

doi:10.1074/jbc.M114.631630.
27. Ichinose, M.; Uchida, M.; Sugita, M. Identification of a pentatricopeptide repeat RNA editing factor in Physcomitrella patens chloroplasts. *FEBS Lett.* **2014**, *588*, 4060–4064, doi:10.1016/j.febslet.2014.09.031.
28. Zhou, W.; Cheng, Y.; Yap, A.; Chateigner-Boutin, A.L.; Delannoy, E.; Hammani, K.; Small, I.; Huang, J. The Arabidopsis gene YS1 encoding a DYW protein is required for editing of rpoB transcripts and the rapid development of chloroplasts during early growth. *Plant J.* **2009**, *58*, 82–96, doi:10.1111/j.1365-313X.2008.03766.x.
29. Yagi, Y.; Tachikawa, M.; Noguchi, H.; Satoh, S.; Obokata, J.; Nakamura, T. Pentatricopeptide repeat proteins involved in plant organellar RNA editing. *RNA Biol.* 2013, *10*, 1419–1425.
30. Pyo, Y.J.; Kwon, K.C.; Kim, A.; Cho, M.H. Seedling lethal1, a pentatricopeptide repeat protein lacking an E/E+ or DYW domain in Arabidopsis, is involved in plastid gene expression and early chloroplast development. *Plant Physiol.* **2013**, *163*, 1844–1858, doi:10.1104/pp.113.227199.
31. Zhang, H.D.; Cui, Y.L.; Huang, C.; Yin, Q.Q.; Qin, X.M.; Xu, T.; He, X.F.; Zhang, Y.; Li, Z.R.; Yang, Z.N. PPR protein PDM1/SEL1 is involved in RNA editing and splicing of plastid genes in Arabidopsis thaliana. *Photosynth. Res.* **2015**, *126*, 311–321, doi:10.1007/s11120-015-0171-4.
32. Tang, J.; Zhang, W.; Wen, K.; Chen, G.; Sun, J.; Tian, Y.; Tang, W.; Yu, J.; An, H.; Wu, T.; et al. OsPPR6, a pentatricopeptide repeat protein involved in editing and splicing chloroplast RNA, is required for chloroplast biogenesis in rice. *Plant Mol. Biol.* **2017**, *95*, 345–357, doi:10.1007/s11103-017-0654-0.
33. Xiao, H.; Xu, Y.; Ni, C.; Zhang, Q.; Zhong, F.; Huang, J.; Liu, W.; Peng, L.; Zhu, Y.; Hu, J. A rice dual-localized pentatricopeptide repeat protein is involved in organellar RNA editing together with OsMORFs. *J. Exp. Bot.* **2018**, *69*, 2923–2936, doi:10.1093/jxb/ery108.
34. Jiang, T.; Zhang, J.; Rong, L.; Feng, Y.; Wang, Q.; Song, Q.; Zhang, L.; Ouyang, M. ECD1 functions as an RNA-editing trans-factor of rps14-149 in plastids and is required for early chloroplast development in seedlings. *J. Exp. Bot.* **2018**, *69*, 3037–3051, doi:10.1093/jxb/ery139.
35. Sun, Y.K.; Gutmann, B.; Yap, A.; Kindgren, P.; Small, I. Editing of chloroplast rps14 by PPR editing factor EMB2261 is essential for Arabidopsis development. *Front. Plant Sci.* **2018**, *9*, doi:10.3389/fpls.2018.00841.
